# Non-Contrast Enhanced MR Angiography (NCE-MRA) of the Calf: A Direct Comparison between Flow-Sensitive Dephasing (FSD) Prepared Steady-State Free Precession (SSFP) and Quiescent-Interval Single-Shot (QISS) in Patients with Diabetes

**DOI:** 10.1371/journal.pone.0128786

**Published:** 2015-06-02

**Authors:** Na Zhang, Liqiu Zou, Yi Huang, Dexiang Liu, Yukuan Tang, Zhaoyang Fan, Hanwei Chen, Xin Liu

**Affiliations:** 1 Lauterbur Research Center for Biomedical Imaging, Shenzhen Institutes of Advanced Technology of Chinese Academy of Sciences, Shenzhen, China; 2 Shenzhen Key Laboratory for MRI, Shenzhen Institutes of Advanced Technology of Chinese Academy of Sciences, Shenzhen, China; 3 Department of Radiology, Peking University Shenzhen Hospital, Shenzhen, China; 4 Department of Radiology, Guangzhou Panyu Central Hospital, Guangzhou, China; 5 Biomedical Imaging Research Institute, Cedars-Sinai Medical Center, Los Angeles, CA, United States of America; University of Washington School of Medicine, UNITED STATES

## Abstract

**Objectives:**

To compare the image quality and diagnostic performance of two non-contrast enhanced MR angiography (NCE-MRA) techniques using flow-sensitive dephasing (FSD) prepared steady-state free precession (SSFP) and quiescent-interval single-shot (QISS) for the calf arteries in patients with diabetes.

**Materials and Methods:**

Twenty six patients underwent the two NCE-MRA techniques followed by contrast-enhanced MRA (CE-MRA) of lower extremity on a 1.5T MR system. Image quality scores, arterial stenosis scores, signal-to-noise ratio (SNR), contrast-to-noise ratio (CNR), vessel sharpness, and diagnostic accuracy for detecting more than 50% arterial stenosis were evaluated and statistically compared using CE-MRA as the reference standard.

**Results:**

All examinations were performed successfully. Of the total 153 calf arterial segments obtained in the 26 patients, FSD and QISS showed no significant difference in the number of diagnostic arterial segments (151 [98%] vs. 147 [96%], respectively, P>0.05). The image quality of FSD was higher than that of QISS in the peroneal artery and posterior tibial artery (P<0.05), but no significant difference in the anterior tibial artery (P>0.05). SNR and CNR of FSD were higher than those of QISS (P<0.01), while FSD showed comparable vessel sharpness compared with QISS (P>0.05). The time efficiency of SNR and CNR between FSD and QISS showed no significant difference when taking into account the times for FSD-related scout scans. There was no difference in sensitivity (95% vs. 93%, P>0.05) and negative predictive value (98% vs. 97%, P>0.05) between FSD and QISS for detecting stenosis greater than 50%. However, FSD showed higher specificities (99% vs. 92%, P<0.05) and diagnostic accuracy (98% vs. 92%, P<0.05) compared to QISS.

**Conclusion:**

Both FSD and QISS had similar high sensitivity and negative predictive value for detecting calf arteries with over 50% stenosis, but FSD showed slightly higher diagnostic specificity and better depiction of arterial lesions due to its isotropic submillimeter spatial resolution. QISS, being an easier to use and less time-consuming technique, could be a method of choice for rapid screening of arterial disease of the lower extremity.

## Introduction

CE-MRA has been widely used for detecting arterial stenosis greater than 50% in the lower extremity [1–3]. However, it has several limitations. High spatial resolution is necessary to depict the small size arteries in the lower extremity. Due to the short contrast first-pass time window in arteries, the spatial resolution is limited by the imaging time. The images are also vulnerable to venous contamination. Enhanced veins commonly seen in CE-MRA of the calf and foot. Dynamic CE-MRA can help reduce venous artifacts, but cannot help improve spatial resolution and more volume of contrast agent is needed. Moreover, the need for gadolinium-based contrast agent can occasionally lead to nephrogenic systemic fibrosis in patients with pre-existing renal insufficiency [4–5]. This is particularly relevant to patients with diabetes. Therefore, a MRA technique for peripheral artery that does not require the use of contrast agent (non-contrast enhanced MRA or NCE-MRA) is highly desired.

Recently, several NCE-MRA techniques have been developed to image arteries of the lower extremity. Turbo spin echo based techniques such as fresh blood imaging [6] and native-SPACE [7] allow depiction of peripheral arteries with large fields of view and reduced venous artifacts [8–10]. However, due to the inherent flow-spoiling effects of turbo spin-echo, the techniques could exhibit signal void at fast and/or turbulent flow that is commonly present distal to stenosis, potentially leading to overestimation of stenosis [11,12].

To achieve higher SNR of arterial blood and less flow artifacts, two techniques based on SSFP, namely FSD [13] and QISS [14], have been proposed recently. FSD uses three-dimensional (3D) acquisition scheme. It allows for isotropic high spatial resolution which helps the visualization of stenosis lesions. The technique requires tune-up of imaging parameters on a case-by-case basis. QISS does not have any patient dependent imaging parameter and is simple to use clinically. However, it is based on two-dimensional (2D) imaging and so its slice resolution is relatively low. Several recent clinical studies showed that both techniques have demonstrated great clinical potential in diagnosing arterial disease in the lower extremities [15–17]. To our knowledge, however, there was no study that directly compares the potential benefits and limitations of the two NCE-MRA techniques in the diagnosis of arterial disease in the lower extremities.

The purpose of this study was to compare the image quality and diagnostic accuracy of the FSD and QISS techniques for the detection of lower extremity arterial stenosis in a diabetic patient cohort, using conventional high resolution CE-MRA as the reference standard.

## Materials and Methods

### Patients

Twenty six patients (16 men, 10 women; mean age, 59 years; age range, 34–79 years) with type II diabetes diagnosed according to 2006 WHO diabetes criteria [18], who were referred for lower extremity CE-MRA, were consecutively recruited in this prospective study. Patients were excluded from the study if they had severe renal failure with a glomerular filtration rate (GFR) < 30 ml/min or general contraindications to MR examination such as claustrophobia and pacemaker. This study was approved by the Peking University Shenzhen hospital institutional review board, and written informed consent was obtained from all patients. A total of 153 arterial segments of the calf were obtained in the 26 patients with 51 legs (one patient had one leg amputated). The mean acquisition times were 3.8 ± 0.5 and 2.6 ± 0.7 (2–3 imaging groups) minutes for FSD and QISS, respectively. However, FSD imaging required additional time for the phase-contrast scan and the first-order moment (m1) scout scan, resulting in a total scan time of 5.1 ± 0.6 minutes.

### Data Acquisition

All MRA studies were performed on a 1.5 T system (MAGNETOM Avanto, Siemens Healthcare, Erlangen, Germany). Patients were placed feet-first-supine in the scanner. Two six-element body coils together with the spine coils were used for signal reception. The coils covered the two bilateral thigh and calf artery. Localizers were first used to define the imaging region. NCE-MRA was first performed. It was immediately followed by CE-MRA. The same coils were used in both examinations. Because calf arteries are smaller than thigh and pelvic arteries, and significant stenoses are often present in the calf arteries in diabetic patients [19], FSD was only performed on calves.

### NCE-MRA: QISS Technique

Images were obtained by using a Siemens work-in-progress QISS sequence, i.e. ECG-gated 2D single shot TrueFISP (SSFP). The sequence set-up followed that in [14]. Eight consecutive groups, each consisting of 60 axial slices, were acquired to cover the lower extremity arteries from the level of the distal aorta to the pedal arteries. The imaging parameters were as follows: ECG triggered, one 2D slice per heartbeat, TR/TE = 708.1 /1.4 ms, echo spacing = 3.4ms, quiescent interval = 228ms, inversion time = 350ms, flip angle = 90°, trigger delay = 100ms, 2.4mm effective slice thickness (3mm with 0.6mm overlap), GRAPPA parallel acceleration factor = 2, bandwidth = 695 Hz/pixel, full Fourier sampling, and fat suppression, matrix = 560×800, field of view (FOV) = 280×400mm (in-plane spatial resolution of 0.5mm). The acquisition time for the calf arteries (2–3 groups) = 1.5–4 min, depending on the heart rate.

### NCE-MRA: FSD Technique

A phase-contrast scan was first performed to determine the systolic and mid-diastolic periods of the patient’s cardiac cycle. The first-order moment (m1) value of the FSD gradients were individually optimized using a scout approach (15~50 mT·ms^2^) [20]. FSD MRA was then performed using ECG-triggered 3D SSFP with FSD magnetization preparation [11]. The arterial images with dramatically suppressed background and venous signals were obtained by subtracting the dark-artery scan acquired at systole from a bright-artery scan acquired at diastole. Imaging parameters for FSD included: TR/TE = 468.4 ms/1.6 ms, echo spacing = 3.5ms, flip angle = 90°, FOV = 400 × 320 × 60–70 mm^3^, voxel size = 0.9 × 0.9 × 0.9 mm^3^, receiver bandwidth = 965 Hz/pixel, parallel acceleration factor rate 2 (GRAPPA) in the phase-encoding direction was used, 60 lines were acquired per heartbeat, acquisition time = 2.5–5.5 min, depending on the heart rate.

### CE-MRA

High-resolution lower extremity CE-MRA from the aortoiliac bifurcation to feet was performed with a standard bolus-chase three-station technique using a 3D gradient-echo FLASH sequence. Imaging parameters included: TR/TE = 3.2 /1.1 ms, flip angle = 25°, FOV = 320 × 320, matrix = 256 × 256, voxel size = 1.2×1.2×1.2 mm^3^, receiver bandwidth = 450 Hz/pixel, GRAPPA acceleration factor = 2. Acquisition time for one 3D dataset was 18–19 seconds. Gd-DTPA (Magnevist, Schering AG, Berlin, Germany) was administered intravenously at a dose of 0.2 mmol/kg body weight using an automated injector (Medrad, Warrendale, PA, USA), followed by an injection of 30 mL saline at the same rate. The injection rate (2 to 2.5 ml/sec) was adjusted to cover 70% of the total acquisition time.

### Data Analysis

Maximum intensity projection (MIP) images of the entire volume and targeted thin slab of the calf arteries were reconstructed with standardized post-processing procedures for both CE-MRA and NCE-MRA on an off-line workstation (Leonardo, Siemens Healthcare, Erlangen, Germany), and were used for assessing image quality and arterial stenosis. In addition, source images were used for quantitative measurement.

Image quality on FSD and QISS scans were evaluated by two experienced radiologists (Y.H. and D.L.) in consensus. Both were blinded to the techniques used and other clinical test information. To reduce potential bias, the FSD and QISS images from the same patient were assessed in random order, with an interval of four weeks in between. Each calf artery was divided into three segments: anterior tibial artery, peroneal artery, and posterior tibial artery. Accordingly six arterial segments for each patient were evaluated and scored with a four-point scale: 1, poor or nondiagnostic arterial display; 2, fair diagnostic arterial display and delineation of the arterial structures with detection of lesions still possible; 3, good diagnostic arterial display without impaired delineation of the vessel structures; and 4, excellent diagnostic arterial display with sharp delineation of artery in full length.

**Fig 1:**
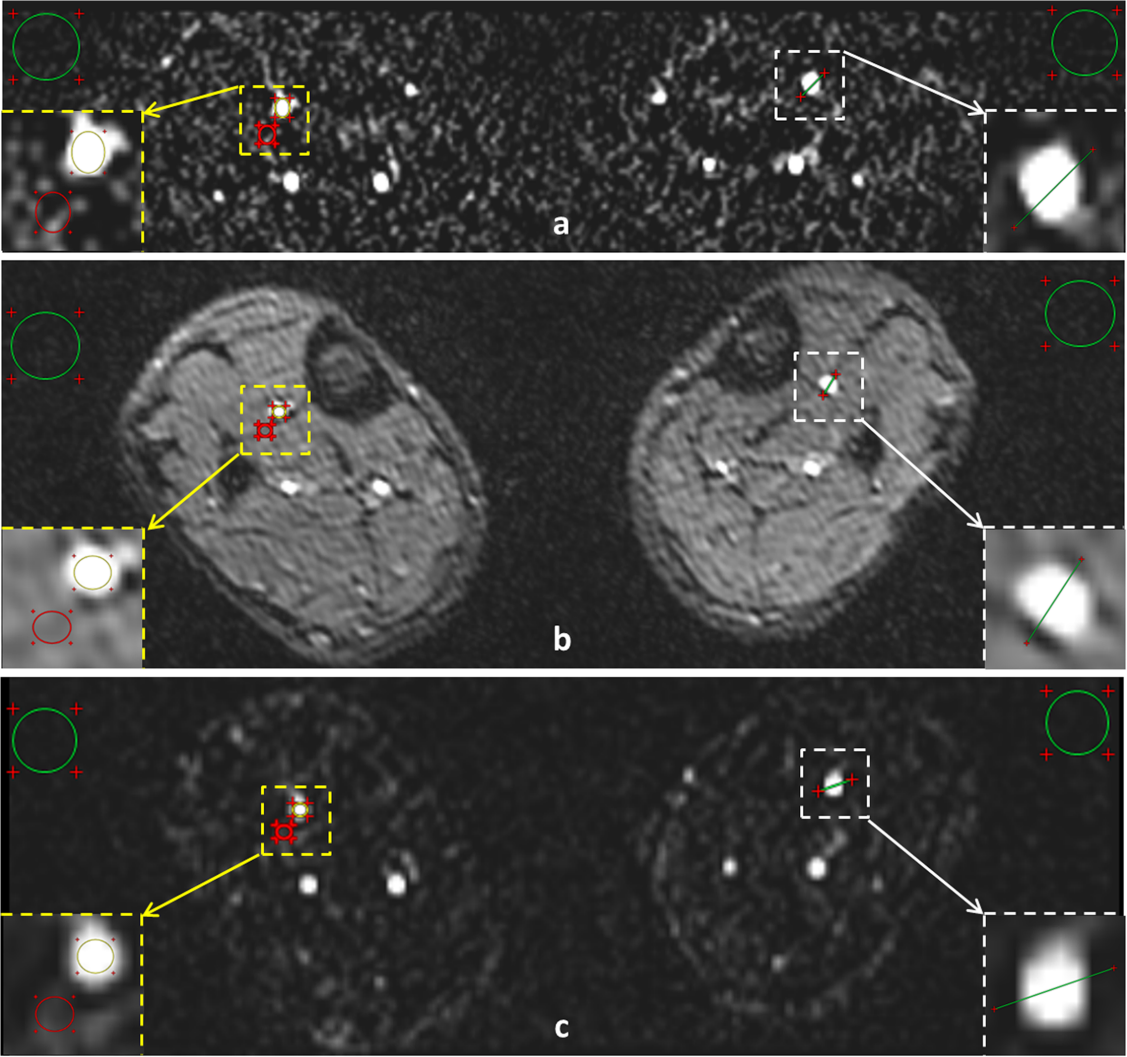
An example image including FSD (a), QISS (b), and CE-MRA (c) from which the SNR, CNR, and vessel sharpness were derived. The circles drawn in the cross-section of arterial segments (yellow circles) and the adjacent tissue (red circles) indicated the representative ROIs for the calculation of signal intensity of arterial blood and tissue, respectively. The circles drawn in the background (green circles) indicated the representative large air ROI for the calculation of noise. The line drawn perpendicularly to the vessel wall indicated the representative user-selected line for the calculation of vessel sharpness.

Quantitative measurements including arterial blood signal-to-noise ratio (SNR), artery-tissue contrast-to-noise ratio (CNR), and vessel sharpness were performed on both NCE-MRA and CE-MRA by one author (N.Z.). SNR was calculated as the average signal intensity within a circular region of interest (ROI area is about 0.02–0.25 cm^2^) selected from the center of arterial segments divided by the mean standard deviation (SD) of the background noise. The CNR was calculated as the mean value of the difference between the signal intensity in the arterial blood within the same ROI ares above and the adjacent tissue divided by the mean SD of the background noise. Due to inherent spatial variations of the noise in the case of parallel imaging, noise was defined as the mean SD of signals in 2 large air ROI (area is about 2.3–6.8 cm^2^ chosen appropriately to avoid areas of phase ghosting at the edge of the FOV [21]. The time efficiency of SNR (SNR _eff_) and CNR (CNR _eff_) of an image set was calculated as SNR and CNR divided by the square root of the acquisition time for that image set respectively [22]. Vessel sharpness was calculated based on a previously described method using a custom MATLAB program [23]. Individual 2D axial original images were magnified four times using bilinear interpolation, and signal intensity profiles were generated along a user-selected lines perpendicular to the vessel wall. On each side of the signal intensity profile, the 20% and 80% points between the maximal wall signal intensity and background signal intensity were identified. The distance between the 2 points was then measured. Sharpness was calculated as the inverse of the averaged value from the 2 widths. The higher this value the better the vessel is delineated. An example image where representative ROIs were marked for SNR and CNR is demonstrated in Fig 1.

The assessment of stenosis severity based on FSD and QISSimages was performed in consensus by two radiologists (Y.H. and D.L.). The percentage stenosis of the vessels based on CE-MRA was performed in consensus by another two radiologists (H.C. and X.L.) with ten years of experience in cardiovascular imaging. FSD and QISS images from the same patient were assessed in random order, with an interval of four weeks in between. A grading system from the American College of Radiology in multi-institutional trial of peripheral MRA was used to evaluate the arterial stenosis degree [24,25]: 0, normal; 1, minimal stenosis of less than 50%; 2, one lesion with 50% or greater stenosis; 3, more than one lesion with 50% or greater stenosis; and 4, occlusion. In addition, overall sensitivity, specificity, positive predictive value, negative predictive value, and accuracy of FSD and QISS for detecting significant stenosis (≥ 50%) were calculated using the CE-MRA as the reference standard. When two or more stenoses were present in one segment, the most severe lesion was used for severity grading. The arterial segments in CE-MRA with nondiagnostic image quality and the corresponding segments on NCE-MRA were excluded from analysis. An intention-to-diagnose approach was used to calculate diagnostic accuracy and nondiagnostic segments on NCE-MRA that were diagnostic on CE-MRA were considered as false [26].

### Statistical Analysis

All statistical analyses were performed using SPSS software (version 19.0, IBM SPSS). Quantitative results of the measurements were presented as mean ± SD. The SNR, CNR, SNR _eff_, CNR _eff_, and vessel sharpness measured from the two NCE-MRA techniques were compared using a paired Student t-test. The stenosis scores and image quality scores for the FSD and QISS were compared by using a nonparametric Wilcoxon signed rank test. For the determination of significant stenosis (≥ 50%), the overall sensitivity, specificity, PPV, NPV, and accuracy of the two NCE-MRA techniques, using CE-MRA as the reference, were estimated on a per-segment basis and compared with a Fisher’s exact test. A *p* value of <0.05 was considered to indicate statistical significance.

## Results

All examinations were performed successfully and were well tolerated by the patients. FSD and QISS showed no significant difference in the number of diagnostic (quality score ≥ 2) arterial segments (151 of 153 segments [98%] vs. 147 of 153 segments [96%] for FSD and QISS, respectively, *p* = 0.15). Among the 2 nondiagnostic arterial segments on FSD, one was due to severe soft tissue signal contamination and the other was due to poor arterial blood SNR. All the 6 nondiagnostic segments on QISS were caused by severe soft tissue signal contamination.

**Fig 2:**
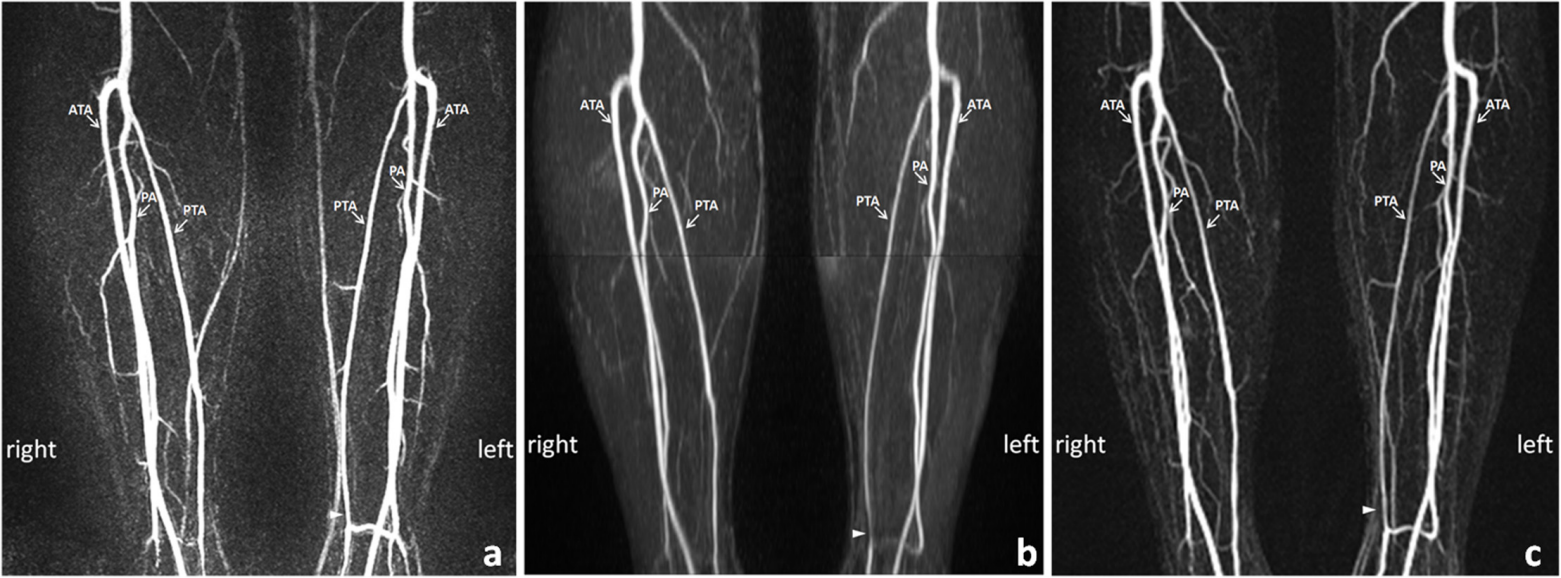
MIP images with the identical window level of FSD (a) and CE-MRA (c) of the calf arteries show no significant stenosis in the bilateral calf arteries in a 54-year-old woman with diabetes. Soft tissue artifacts and a false stenosis caused by signal loss are seen at the left distal peroneal artery (PA) (arrowhead) on the MIP image of QISS (b), of which the optimal window level is different from that of FSD and CE-MRA. MIP: maximum intensity projection; FSD: flow-sensitive dephasing; CE-MRA: contrast-enhanced MR angiography; QISS: quiescent-interval single-shot; ATA: anterior tibia artery; PTA: posterior tibia artery.

**Fig 3:**
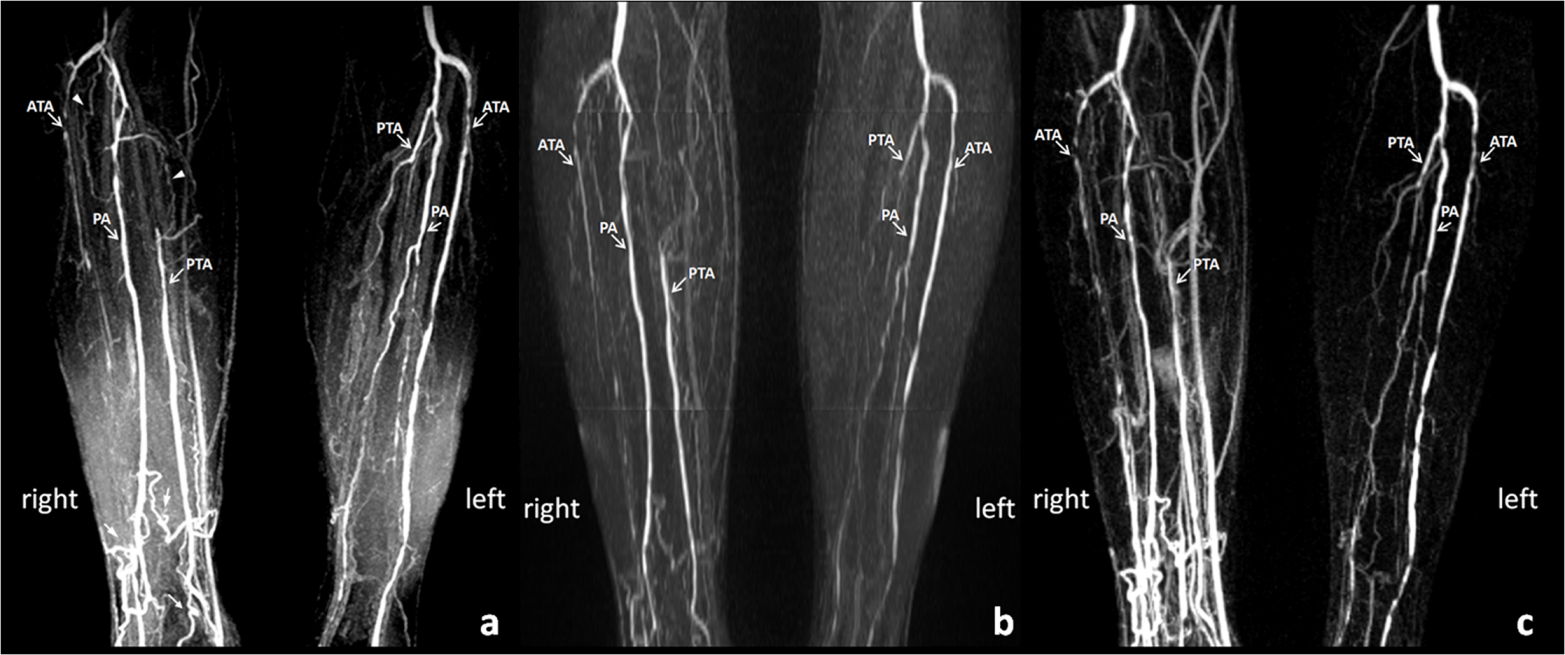
MIP images with different optimal window level of FSD (a), QISS (b), and CE-MRA (c) of the calf arteries demonstrate diffused severe lesions in the left anterior tibia artery (ATA) and occlusions in the right ATA and left posterior tibia artery (PTA) in a 65-year-old man with diabetes and renal insufficiency. Collaterals are seen at the right proximal peroneal artery and PTA (arrow head) as well as the distal calf arteries (arrow). FSD shows better delineation of the arterial lesions and small collaterals compared to QISS. Soft tissue and deep venous contaminations caused by faster deep venous flow appear on the FSD image. But it doesn’t affect the evaluation of arterial lesions because of the high arterial contrast as well as isotropic spatial resolution. MIP: maximum intensity projection; FSD: flow-sensitive dephasing; QISS: quiescent-interval single-shot; CE-MRA: contrast-enhanced MR angiography; PA: peroneal artery (PA).

The comparison of image quality between FSD and QISS is detailed in Table 1. The image quality of FSD was higher than that of QISS in the peroneal artery (3.5 ± 0.4 vs. 3.3 ± 0.8, *p* = 0.004) and posterior tibial artery (3.5 ± 0.6 vs. 3.4 ± 0.7, *p* = 0.02), but no significant difference was observed in the anterior tibial artery (3.4 ± 0.6 vs. 3.2 ± 0.8, *p* = 0.07). The number of different kinds of artifacts for FSD and QISS is detailed in Table 2. Soft tissue and superficial venous contaminations were the main cause of the lower image quality with QISS. There were noticeable residual signals from superficial veins (33 of 51, 65%), soft tissue (39 of 51, 76%), deep veins (2 of 51, 4%), and motion (3 of 51, 6%). For FSD, soft tissue and deep venous contaminations mainly affect the image quality. There were noticeable residual signals from superficial veins (25 of 51, 49%), soft tissue (33 of 51, 65%), deep veins (35 of 51, 69%), and motion (2 of 51, 4%). Figs 2 and 3 demonstrate the higher spatial resolution and SNR in FSD images in depicting calf arteries and detecting arterial stenosis.

**Table 1:** Comparison of image quality of three calf arterial segments for NCE-MRA in 26 patients with diabetes.

	Anterior tibial artery	Peroneal artery	Posterior tibial artery	All segments
**FSD**	3.37 ± 0.62	3.55 ± 0.46	3.58 ± 0.58	3.50 ± 0.56
**QISS**	3.23 ± 0.82	3.30 ± 0.81	3.43 ± 0.74	3.32 ± 0.79
***p*** ^**a**^	0.067	0.004	0.020	< 0.001

^a^Wilcoxon signed rank test.

**Table 2:** The number of different kinds of artifacts for NCE-MRA in 26 patients with diabetes.

	Soft tissue contaminations	Superficial venous contaminations	Deep venous contaminations	Motion artifacts
**FSD**	65% (33 of 51)	49% (25 of 51)	69% (35 of 51)	4% (2 of 51)
**QISS**	76% (39 of 51)	65% (33 of 51)	4% (2 of 51)	6% (3 of 51)

**Fig 4:**
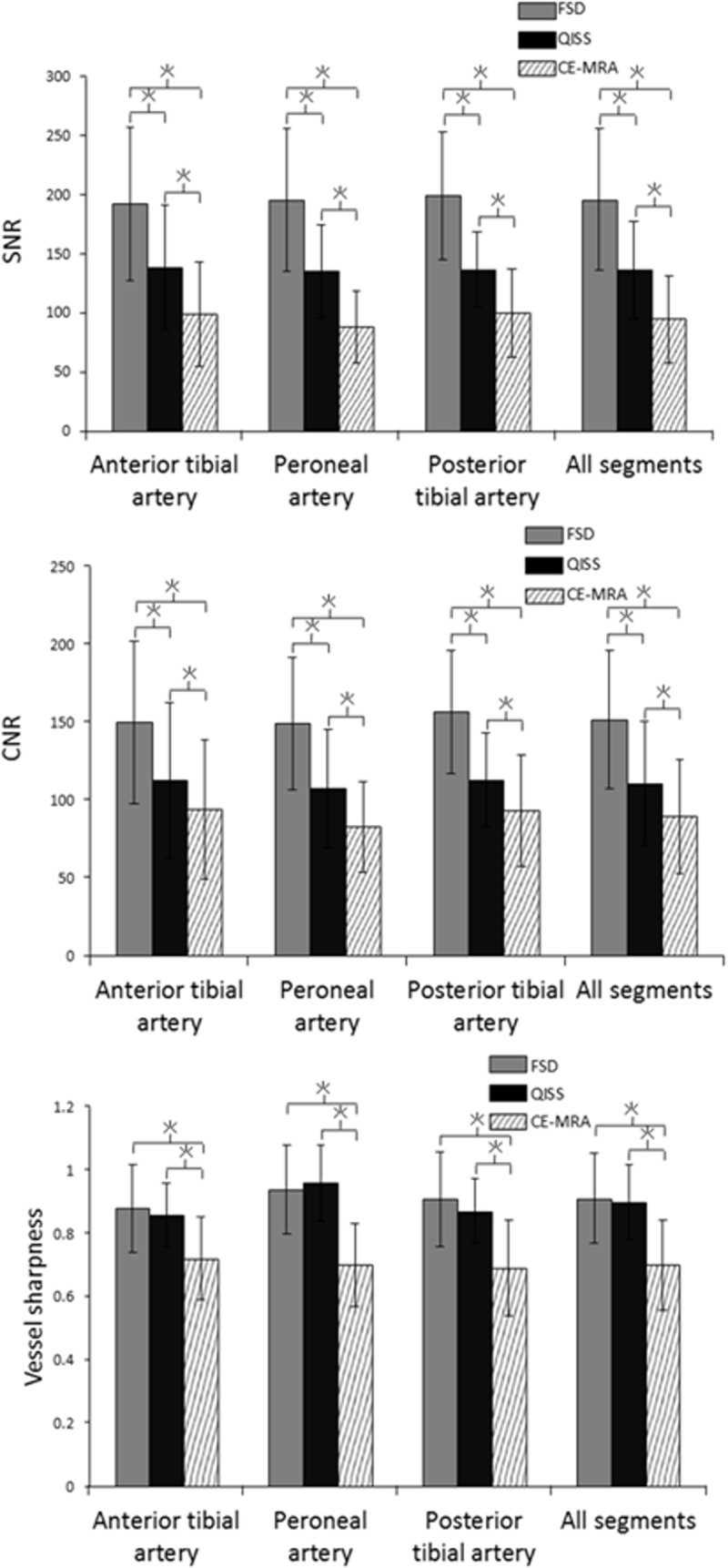
Comparison of SNR, CNR, and vessel sharpness between FSD, QISS, and CE-MRA in three arterial segments of the calf. Each column represents average measurements and error is shown as standard deviation. Asterisks indicated significant difference (P < 0.05). SNR: signal-to-noise ratio; CNR: contrast-to-noise ratio; FSD: flow-sensitive dephasing; QISS: quiescent-interval single-shot; CE-MRA: contrast-enhanced MR angiography.

**Fig 5:**
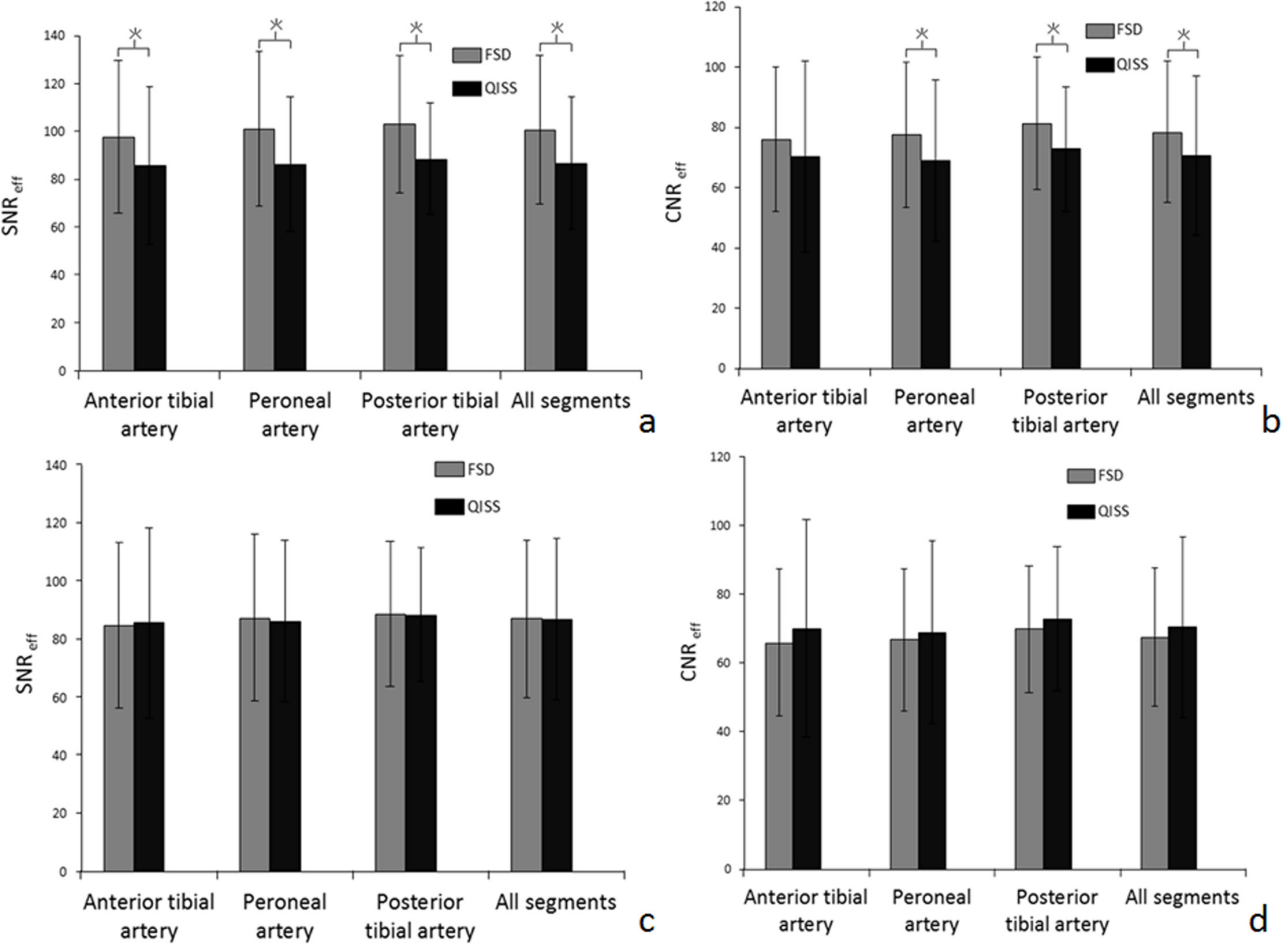
Comparison of the time efficiency of SNR (SNR _eff_) and CNR (CNR _eff_) between QISS and FSD with only dark and bright blood scan (a, b) or adding the phase-contrast and m1-scout scan (c, d)) in three arterial segments of the calf. Each column represents average measurements and error is shown as standard deviation. Asterisks indicated significant difference (P < 0.05). SNR: signal-to-noise ratio; CNR: contrast-to-noise ratio; QISS: quiescent-interval single-shot; FSD: flow-sensitive dephasing.

Comparison of SNR, CNR, and vessel sharpness of calf arterial segments among FSD, QISS, and CE-MRA (reference standard) are illustrated in Fig 4. Quantitative analysis showed that FSD had higher SNR and CNR (196 ± 58 vs. 137 ± 41, *p* < 0.01 and 152 ± 47 vs. 111 ± 32, *p* < 0.01 for SNR and CNR, respectively), but similar vessel sharpness (0.92 ± 0.05 vs. 0.89 ± 0.06, *p* = 0.58) compared to QISS. When compared to CE-MRA, both FSD and QISS had higher SNR, CNR, and vessel sharpness (p<0.05). Fig 5 illustrates how SNR _eff_ and CNR _eff_ compared between FSD and QISS. When considering only dark and bright blood scan times, FSD showed higher time efficiency. When the scan time for phase-contrast and m1-scout scans needed in FSD were taken into account, FSD showed no significant difference compared with QISS in terms of SNR _eff_ and CNR _eff_.

The comparison of stenosis scores between FSD, QISS, and CE-MRA is detailed in Table 3. There was no significant difference between any pair of the three techniques (FSD: 0.89 ± 1.40, QISS: 0.93 ± 1.39, and CE-MRA: 0.85 ± 1.35, all *p* values >0.05).

**Table 3:** Comparison of stenosis scores of three calf arterial segments between NCE-MRA and CE-MRA techniques in 26 patients with diabetes.

	Anterior tibial artery	Peroneal artery	Posterior tibial artery	All segments
**FSD**	1.25 ± 1.63	0.49 ± 0.88	0.92 ± 1.50	0.89 ± 1.40
**QISS**	1.31 ± 1.66	0.47 ± 0.85	1.00 ± 1.41	0.93 ± 1.39
**CE-MRA**	1.22 ± 1.61	0.45 ± 0.87	0.88 ± 1.38	0.85 ± 1.35
***p*** ^**a**^				
**FSD vs. QISS**	1.000	0.257	0.417	0.976
**FSD vs. CE-MRA**	0.157	0.414	0.577	0.177
**QISS vs. CE-MRA**	0.157	0.564	0.096	0.109

^a^Wilcoxon signed rank test.

On CE-MRA, only one arterial segment (anterior tibial artery) was of nondiagnostic image quality due to severe venous contamination caused by arteriovenous fistula. Of the 152 diagnostic segments on CE-MRA images, 42 segments were identified to have more than 50% stenosis. Among the 42 segments, 40 and 39 of them were identified on FSD and QISS images, respectively (sensitivity was 95% and 93% respectively, *p* = 1.0). Of the remaining 110 arterial segments that are either normal or have no significant stenosis on CE-MRA, 2 and 4 nondiagnostic segments on FSD and QISS images were considered to have a significant stenosis according to the intention-to-diagnose analysis respectively. Thus, 108 and 104 segments were correctly identified on FSD and QISS images, respectively (specificity was 98% and 94% respectively, *p* = 0.04). Fig 2 demonstrates a false more than 50% stenosis at the left distal peroneal artery (arrowhead) caused by signal loss in QISS. The comparison of positive predictive value, negative predictive value, and accuracy between FSD and QISS were 95% vs. 86% (*p* = 0.02), 98% vs. 97% (*p* = 0.68), and 97% vs. 94% (*p* = 0.045), respectively.

## Discussion

This single-center clinical study demonstrated that both FSD and QISS can provide satisfactory image quality (high SNR and CNR) for the delineation of calf arteries, and comparable diagnostic performance for the evaluation of arterial stenosis without the use of gadolinium-based contrast agent using CE-MRA as the reference standard. Both NCE-MRA techniques have high negative predictive value for detecting significant stenosis, suggesting that the techniques could be a reliable screening tool for excluding infragenual significant arterial stenosis. Compared to QISS, FSD showed better depiction of small collaterals and higher specificity for assessing the severity of arterial stenosis, likely due to its isotropic submillimeter spatial resolution and higher arterial blood SNR/CNR. On the other hand, QISS is easier to use and less time-consuming as there is no needs for prescribing the imaging volume or scouting for optimal imaging parameters related to the black blood acquisition.

FSD showed significantly higher image quality than QISS except for the anterior tibial artery segments. This may be related to the three-dimension acquisition and isotropic resolution of FSD, which is favorable for the depiction of the smaller segments of peroneal artery and posterior tibial artery. In our study, the main factors affecting image quality of FSD were signal contamination of superficial veins due to fast venous flow, deep veins and soft tissues arising primarily from the signal difference between bright-artery and dark-artery measurements. However, these artifacts do not have significant impact on the evaluation of arterial lesion because of the high SNR and CNR of the arteries acquired by FSD. For QISS images, all segments of nondiagnostic image quality were caused by severe soft tissue signal contamination. This may be related to the use of a constant trigger delay and quiescent interval in all patients. It is anticipated that some patients may have a delayed onset or prolonged duration of systole, thus an individual-tailored trigger delay and quiescent interval might improve the image quality of QISS. In addition, compared with CE-MRA, both FSD and QISS demonstrated significantly improved SNR, CNR, and vessel sharpness in calf arteries. This may be attributed to the use of SSFP in image acquisition which has inherently high blood signal [27]. Fig 2 illustrates the reformatted calf artery images of FSD, QISS, and CE-MRA. With the high SNR, CNR, and vessel sharpness, NCE-MRA demonstrated excellent performance for the delineation of luminal narrowing that were consistent with CE-MRA. Soft tissue and deep venous contaminations caused by faster deep venous flow appeared on the FSD image. However, it did not have significant impact on the evaluation of arterial lesions because of the high arterial contrast as well as isotropic spatial resolution [28].R2-3R2-2R1-9Including the arterial segments of nondiagnostic image quality that were diagnostic on CE-MRA, both FSD and QISS showed a slightly higher negative predictive value for detecting significant stenosis compared to previous report [6]. This may be attributed to the fact that the subjects had more severe arterial lesions in this study. Being a user-friendly and time-efficient run-off MRA technique, QISS could be a method of choice for rapid screening of arterial diseases of the lower extremity in diabetic patients. However, FSD showed higher specificity for assessing severity of arterial stenosis and better delineation of calf arteries, especially for the small tortuous collaterals due to its isotropic submillimeter spatial resolution.

In our study, QISS showed a slightly higher mean stenosis scores than FSD and CE-MRA, although there were no statistically significant difference (0.93 vs. 0.89 and 0.85, respectively, all *p* values > 0.05). This suggested that QISS may tend to overestimate arterial stenosis. Such overestimation may be caused by either a markedly reduced flow velocity or spin dephasing over a relatively large voxel in case of turbulent flow.

This study has several limitations. First, the number of patients was relatively small and conventional x-ray angiography of lower extremity was not available. A multicenter study would be needed to validate the diagnostic accuracy of NCE-MRA for the detection of significant stenosis in the calf arteries. Second, NCE-MRA was acquired prior to CE-MRA in all patients. As patient motion is usually observed towards the end of the examination, the order of image acquisition might have biased the results on subjective image quality. Finally, consensus reading rather than independent reading was performed to determine the degree of stenosis on NCE-MRA. Interobserver agreement was not assessed in the study.

In conclusion, Both FSD and QISS showed high sensitivity and negative predictive value for detecting more than 50% stenosis of calf arteries using CE-MRA as a reference standard. Compared to QISS, FSD had slightly higher specificity and better depiction of calf arteries due to its isotropic submillimeter spatial resolution and higher arterial SNR/CNR. QISS is easier to set up and could be an appropriate choice for rapid screening of arterial disease of lower extremity without use of contrast agents.

## Supporting Information

S1 File The participant-level data in an excel file, which includes individual data of image quality scores, arterial stenosis scores, arterial blood signal-to-noise ratio (SNR), artery tissue contrast-to-noise ratio (CNR), vessel sharpness, the time efficiency of arterial blood SNR (SNR_eff_) and artery tissue CNR (CNR_eff_) with dark and bright blood scan or adding the phase-contrast and m1-scout scan. (XLS)
